# Identification of ferroptosis-related genes and pathways in diabetic kidney disease using bioinformatics analysis

**DOI:** 10.1038/s41598-022-26495-2

**Published:** 2022-12-30

**Authors:** Dezhen Liu, Wei Zhou, Li Mao, Zhaohui Cui, Shanshan Jin

**Affiliations:** 1 Department of Endocrinology, Huai’an First People’s Hospital Affiliated to Nanjing Medical University, Huai’an, Jiangsu China; 2 Department of Endocrinology, Huai’an 82 Hospital, Huai’an, Jiangsu China

**Keywords:** Computational biology and bioinformatics, Diabetes complications

## Abstract

Diabetic kidney disease (DKD) is a major public health issue because of its refractory nature. Ferroptosis is a newly coined programmed cell death characterized by the accumulation of lipid reactive oxygen species (ROS). However, the prognostic and diagnostic value of ferroptosis-related genes (FRGs) and their biological mechanisms in DKD remain elusive. The gene expression profiles GSE96804, GSE30566, GSE99339 and GSE30528 were obtained and analyzed. We constructed a reliable prognostic model for DKD consisting of eight FRGs (SKIL, RASA1, YTHDC2, SON, MRPL11, HSD17B14, DUSP1 and FOS). The receiver operating characteristic (ROC) curves showed that the ferroptosis-related model had predictive power with an area under the curve (AUC) of 0.818. Gene functional enrichment analysis showed significant differences between the DKD and normal groups, and ferroptosis played an important role in DKD. Consensus clustering analysis showed four different ferroptosis types, and the risk score of type four was significantly higher than that of other groups. Immune infiltration analysis indicated that the expression of macrophages M2 increased significantly, while that of neutrophils and mast cells activated decreased significantly in the high-risk group. Our study identified and validated the molecular mechanisms of ferroptosis in DKD. FRGs could serve as credible diagnostic biomarkers and therapeutic targets for DKD.

## Introduction

DKD also called diabetic nephropathy (DN), is the most common cause of end-stage renal disease (ESRD) as a refractory chronic microvascular complication of diabetes mellitus (DM). The main pathological features of DKD are tubular atrophy and tubulointerstitial fibrosis^1^. Currently, only angiotensin converting enzyme inhibitors (ACEI), angiotensin receptor blockers (ARB), and sodium-glucose cotransporter-2 inhibitors (SGLT2i) have been proven to provide partial renoprotection in the progression of DKD^2^. As current treatments for DKD are limited, it is difficult to inhibit DKD from progressing to ESRD, which has become a conundrum for nephrologists and endocrinologists^3^. Therefore, more sensitive biomarkers for early diagnosis, intervention, better classification, and management of DKD are needed.

Ferroptosis is a recently identified atypical form of programmed cell death induced by oxidative stress damage^4^. The main feature of ferroptosis is excessive accumulation of oxidation products. Some studies have found that ferroptosis may promote the progression of DKD in main three ways. First, excessive ROS accumulation cause significant injuries to the kidney. The lipid peroxidation products were found increased in DKD model, like MDA and 4-HNE. Second, reduced antioxidant capacity leads to massive damage to renal tubular epithelial cells. Gpx4, an antioxidant enzyme that reduces phospholipid hydroperoxide, is significantly reduced in DKD and is considered one of the biomarkers of ferroptosis. Third, the imbalance of iron homeostasis leads to iron deposition, the expression of TFR-1 was significantly increased, while the expression of FTH-1 was reduced which means the overload of iron. All of them reveal that ferroptosis may occupy an important position in the progression of DKD^5^. Some studies on DKD showed that xCT and GPX4 were decreased in diabetic kidney biopsy samples which are the key mediators of ferroptosis^6^. The ferroptosis markers including ACSL4, GPX4, iron level and lipid peroxidation products were found significantly changed in streptozotocin induced DKD mice models^7^. In db/db mice model ferroptosis could aggravate the damage of renal tubules through HIF-1α/HO-1 pathway^8^. Some non-coding RNAs are also involved in ferroptosis in DKD^9^. A number of studies have investigated ferroptosis, however, the specific mechanisms of ferroptosis caused by iron and ROS are still unknown. And the regulatory mechanism of ferroptosis in tubular cells has not been fully clarified. Because the coexist of multiple cell death modes in DKD, including ferroptosis, autophagy and pyroptosis, it is an urgent direction to elucidate the specific involvement of ferroptosis in DKD. At present, there are no specific biomarkers of ferroptosis to diagnose and treat DKD until now.

To date, few studies have used bioinformatics methods to improve the understanding of genes related to ferroptosis in DKD. Hu et al.^10^ obtained diabetic nephropathy and normal kidney samples from GSE96804 dataset. Six hub genes (FPR3, C3AR1, CD14, ITGB2, RAC2 and ITGAM) related to ferroptosis in DKD were analyzed. However, this study had small samples from a single microarray analysis, which may have resulted in a high false‐positive rate. Therefore, it is necessary to identify new prognostic ferroptosis markers for the diagnosis and treatment of DKD using comprehensive bioinformatics analyses.

We integrated four microarray datasets from the Gene Expression Omnibus (GEO) database to identify the significant differentially expressed genes (DEGs). The least absolute shrinkage and selection operator (LASSO) regression was used to construct and verify the diagnostic model. To further explore the biological processes of DKD, we conducted a comprehensive enrichment analysis using gene ontology (GO) database, Kyoto encyclopedia of genes and genomes (KEGG) pathway database, gene set enrichment analysis (GSEA), gene set variation analysis (GSVA), and single sample GSEA (ssGSEA) methods. Weighted gene co-expression network analysis (WGCNA) was used to identify co-expressed gene modules and explore the core genes in the network. As the immune system plays an important role in the progression of DKD, we used CIBERSORT to conduct the immune infiltration analysis. Our study establishes a comprehensive network of FRGs related to DKD, providing useful evidence for identifying the role of ferroptosis in the diagnosis and targeted therapy of DKD.

## Results

### Differentially expressed genes

**Figure 1:**
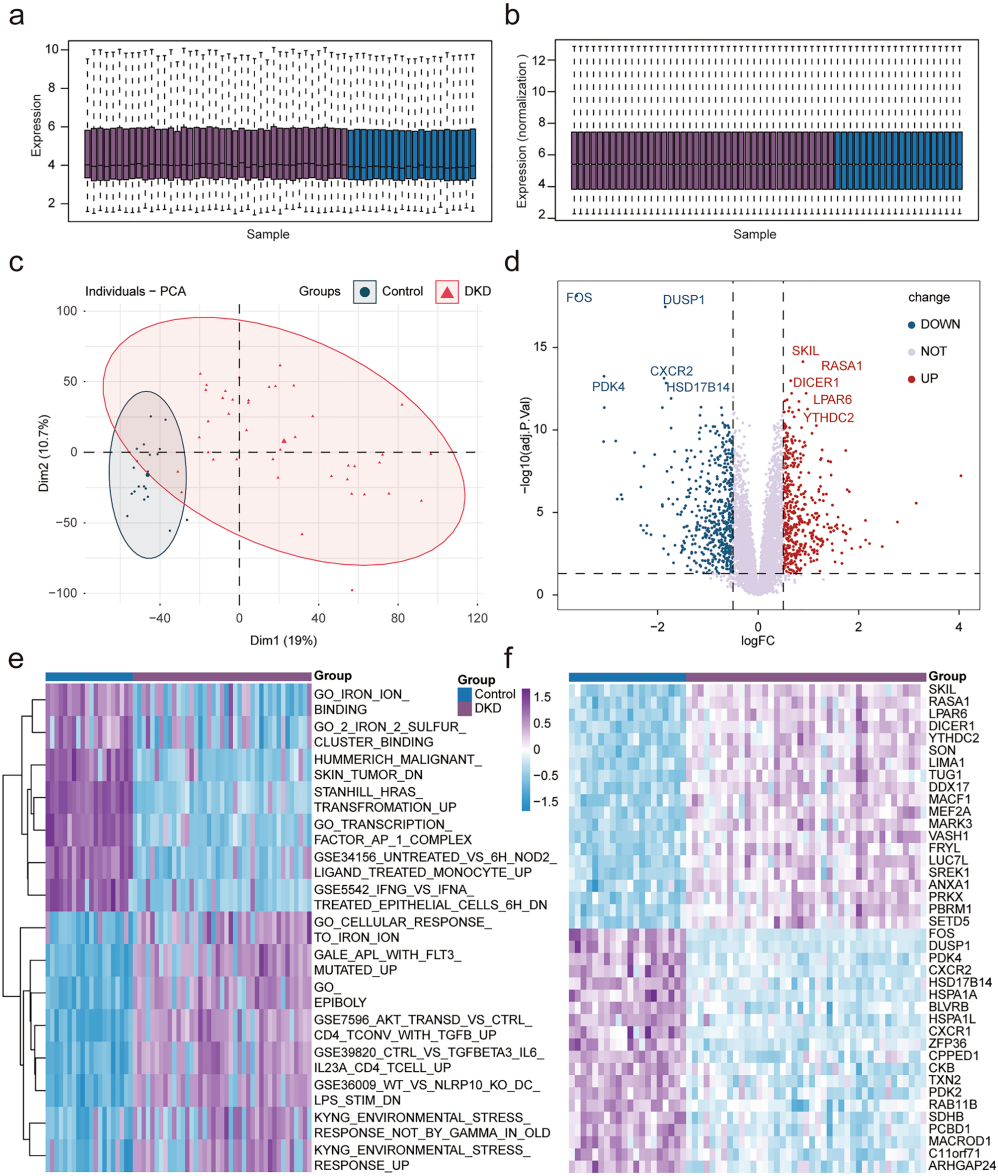
Differentially expressed genes. (**a**) Histogram of GSE96804 data set sample expression value, purple represents DKD samples, dark blue represents normal kidney tissue samples (Control). (**b**) Histogram of GSE96804 data set sample expression value after normalized by limma packets, purple represents DKD samples, dark blue represents normal kidney tissue samples (Control). (**c**) Principal component analysis, red represents DKD samples and light green represents normal kidney tissue samples (Control). (**d**) Volcano map of DEGs. The horizontal axis is logFC and the vertical axis is −log10(adj. *p*). The dotted lines in the figure represent |logFC|> 0.5 and adj. *p* < 0.01 respectively. Red represents up-regulated genes, blue represents down-regulated genes, and the top five genes’ tags are displayed in adj. *p* arrangement. (**e**) Heat map shows the difference of pathway enrichment between the two groups by GSVA, and the top 10 are selected by adj. *p* arrangement for visualization. (**f**) Heat map of the DEGs. Purple represents DKD samples, dark blue represents normal kidney tissue samples (Control), and the top 20 genes are visualized by adj. *p* arrangement. (adj. *p* < 0.05 was considered statistically significant).

The DKD dataset GSE96804(normal group 20; DKD group 41) from the public data platform was downloaded and homogenized to further study the characteristics of DKD and explore efficient markers for early diagnosis and treatment (Fig. 1a,b). Principal component analysis (PCA) showed that the expression profile of normal kidney tissue was significantly different from that of the DKD group (Fig. 1c). DEGs were analyzed between the two groups using the limma package, including 516 upregulated and 441 downregulated genes. The results were visualized using volcano and heat maps (Fig. 1d–f). The abnormal expression difference between the DKD group and the normal group may provide clues for the occurrence and development mechanism of the disease and new treatment methods.

**Figure 2:**
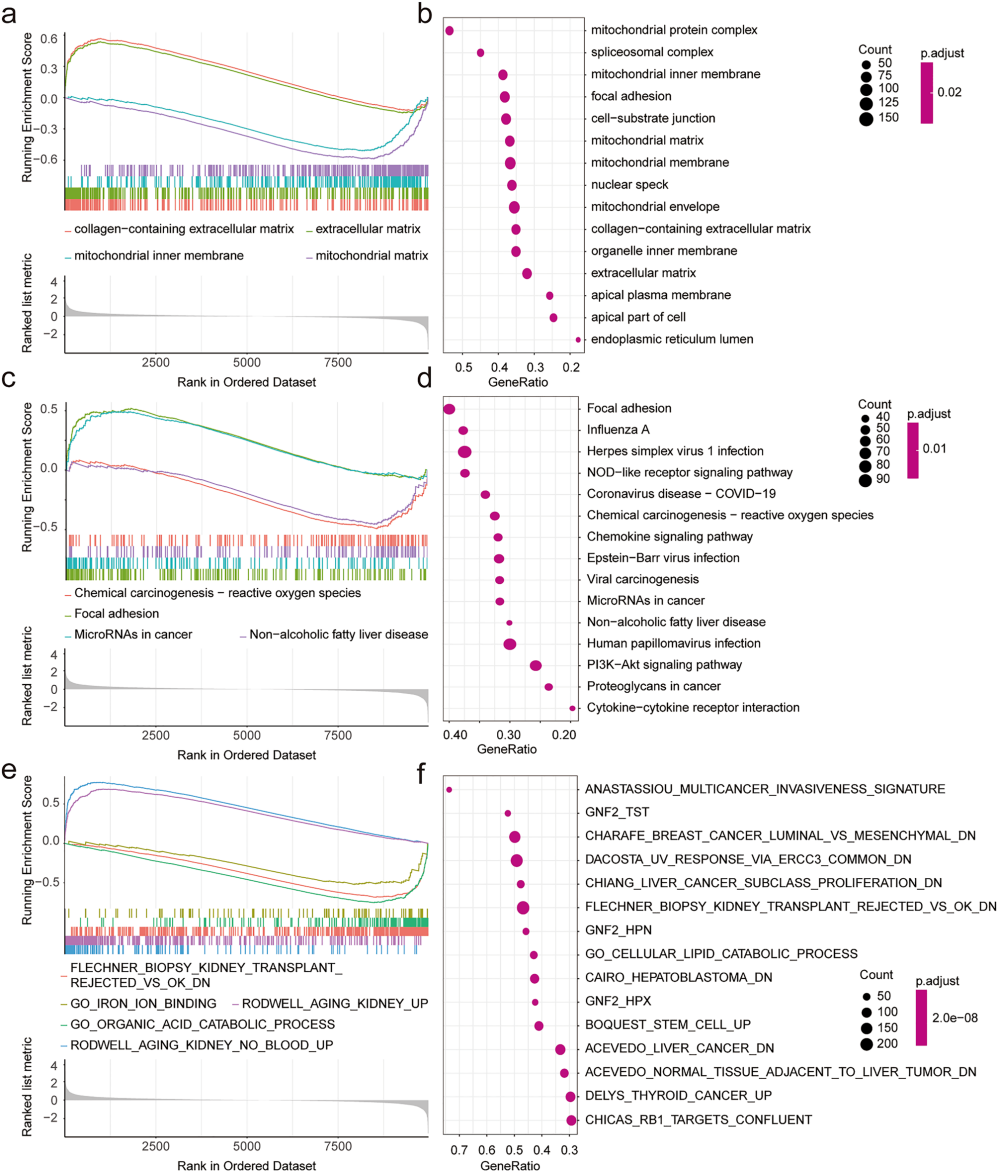
Function analysis. (**a**) The GSEA enrichment method was used to annotate the GO gene function of the DEGs, and the two items with the highest and lowest enrichment scores are visualized according to the enrichment scores. (**b**) The GSEA enrichment method was used to annotate the GO gene function of the DEGs, and the top 15 are visualized according to the enrichment scores. (**c**) The KEGG pathway enrichment analysis of DEGs was carried out by GSEA enrichment method, and the two items with the highest and lowest enrichment scores are visualized according to the arrangement of enrichment scores. (**d**) The KEGG pathway enrichment analysis of DEGs was carried out by GSEA enrichment method, and the top 15 are visualized according to the enrichment score. (**e**) The pathway enrichment analysis of DEGs was carried out according to the MSigDb dataset by the GSEA enrichment method, and the two items with the highest and lowest enrichment scores and ferroptosis related pathway are visualized according to the arrangement of enrichment scores. (**f**) The pathway enrichment analysis of DEGs was carried out according to the MSigDb dataset by GSEA enrichment method, and the top 15 are visualized according to the enrichment score. (adj. *p* < 0.05 was considered statistically significant).

**Figure 3:**
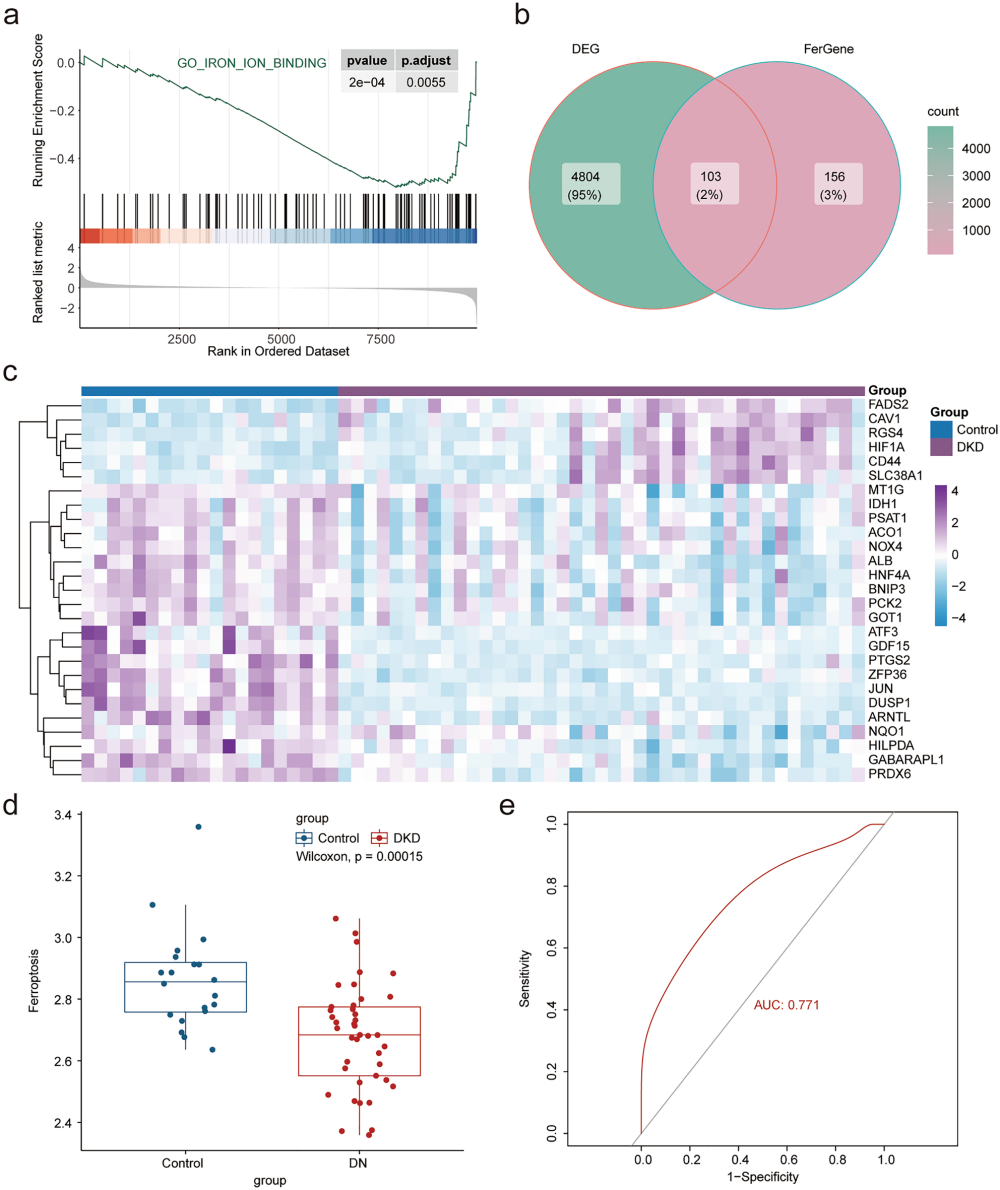
Diagnostic value of FRGs. (**a**) Ferroptosis related pathway (GO_IRON_ION_BINDING) GSEA enrichment analysis. (**b**) Veen map shows the common genes of DEGs and FRGs. (**c**) The heat map shows the differential expression of genes related to ferroptosis. Purple represents the DKD samples and dark blue indicates the normal kidney tissue samples (Control). (adj. *p* < 0.05 was considered statistically significant). (**d**) The box chart shows the difference in the enrichment scores of FRGs between the two groups based on ssGSEA analysis, with blue representing normal group (Control) and red representing DKD group (*p* < 0.05 was considered statistically significant). (**e**) ROC curve shows that FRGs had good diagnostic value in DKD (AUC = 0.771).

Through gene pathway enrichment analysis, we can preliminarily analyze the biological processes or signal pathways that may be involved in the occurrence and development of DKD. We analyzed the enrichment scores of related data sets in the MSigDb database of all samples using GSVA and screened the differential enrichment pathways using the limma package with the filter criteria of |logFC| > 0, adj. *p* < 0.01 (see Supplementary Table S1 online). We found significant differences in multiple signaling pathways between the two groups, which were visualized using a heat map (Fig. 1e). Among the differential signaling pathways, we found that there were differences in several iron ion-related pathways between the two groups, and the differences were statistically significant. To further explore the differences of function and pathway between DKD and normal groups, we used GSEA to perform enrichment analysis on the differential gene list ranked from high to low in the logFC of the GO, KEGG, and MSigDb pathway-related datasets (see Supplementary Tables S2, 3 online). The results showed that the collagen-containing extracellular matrix and extracellular matrix were mainly enriched using GO enrichment analysis, while the mitochondrial inner membrane and mitochondrial matrix (Fig. 2a,b) were inhibited. In the pathway enrichment analysis, focal adhesion and microRNAs in cancer were enriched, while chemical carcinogenesis-reactive oxygen species and non-alcoholic fatty liver disease were inhibited (Fig. 2c,d). Multiple pathways were enriched in the MSigDB-related dataset enrichment analysis, ferroptosis pathway enrichment was inhibited, and the results were consistent with GSVA (Fig. 2e,f). GSEA enrichment analysis of ferroptosis related pathways in DKD were visualized in Fig. 3a. Ferroptosis is a new type of programmed cell death, few studies have found the role of ferroptosis in the development of DKD. In order to further explore the potential relationship between ferroptosis and DKD, 382 FRGs were downloaded from the FerrDb. The expression of 103 genes was significantly different between the normal and DKD groups (Fig. 3b). The differentially expressed genes related to ferroptosis with |logFC| > 0.5 were shown by heat map (Fig. 3c). To further confirm our conclusion, we used the ssGSEA algorithm to calculate the ferroptosis gene enrichment score of each sample based on FRGs. We found that the degree of ferroptosis enrichment in the DKD group was significantly lower than that in the normal group, which was consistent with the GSEA and GSVA results. At the same time, taking the enrichment score of ferroptosis as a predictive index, ROC curve proved that the ferroptosis-related gene set had a significant diagnostic value (AUC:0.771) (Fig. 3d,e). The results suggested that there was a significant difference between the normal and DKD groups at the transcriptional level, and the ferroptosis-related pathways were significantly changed in DKD. We believe that ferroptosis may plays a certain role in the occurrence and development of DKD. FRGs may be used as new predictive biomarkers to provide new angle for the diagnosis and treatment of DKD.

### Construction and verification of prognostic model of FRGs

**Figure 4:**
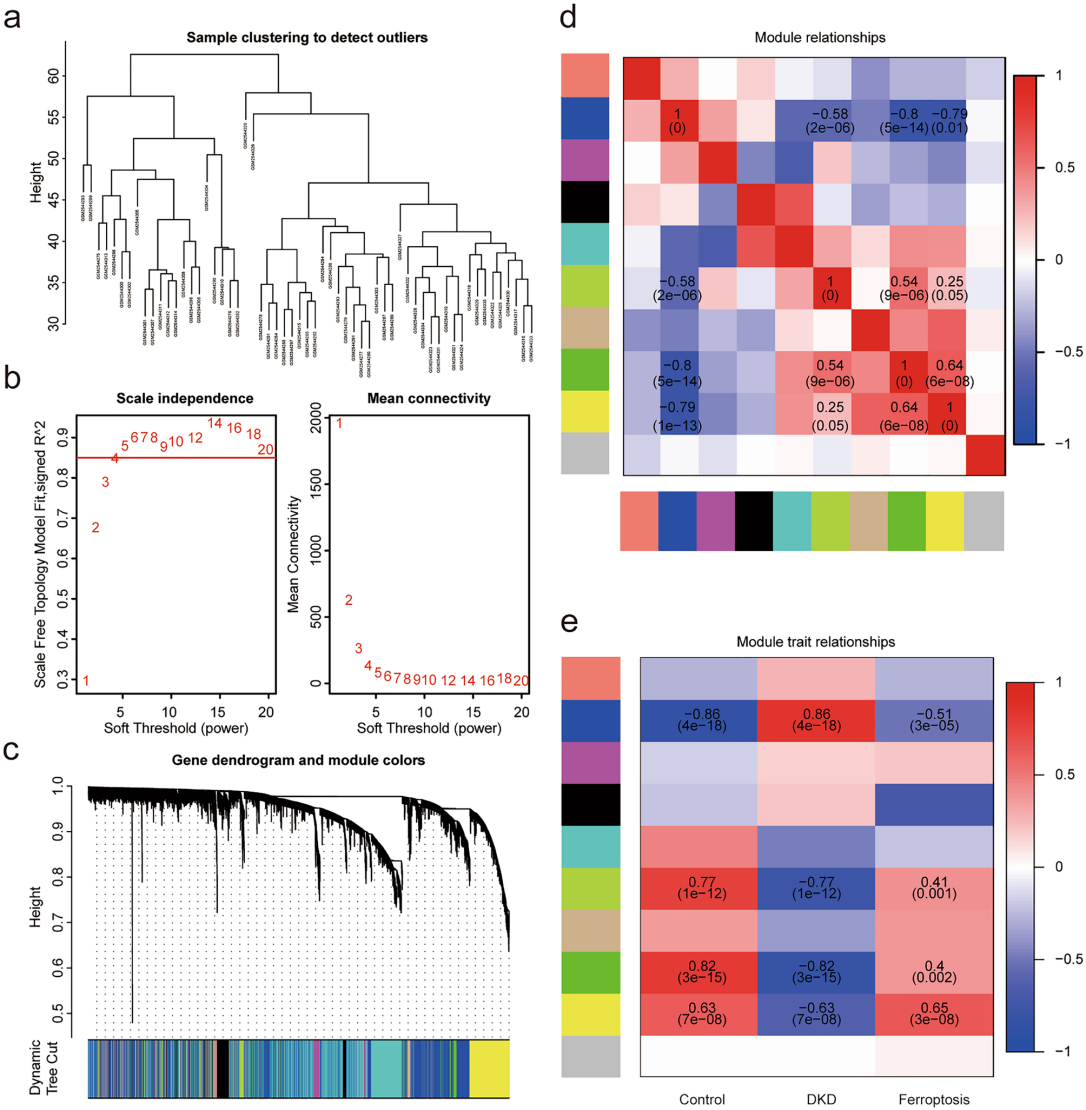
WGCNA recognize and diagnose the ferroptosis related gene set. (**a**) After removing two samples with great differences according to stagger clustering, the tree view is displayed. (**b**) Construction of WGCNA gene module, the results suggest that there are 10 gene modules, which are represented by different colors, and gray represents the unclassified genes. (**c**) Build a scale-free network with five as the best soft threshold. (**d**) The correlation analysis between modules, red represents positive correlation and blue represents negative correlation. (**e**) The correlation analysis between modules and clinical features. Red represents positive correlation and blue represents negative correlation. Blue module (3788) is significantly positively correlated with DKD and negatively correlated with ferroptosis, while green module (3788), greenyellow module (114) and yellow module (1237) are just the opposite (*p* < 0.05 was considered statistically significant).

**Figure 5:**
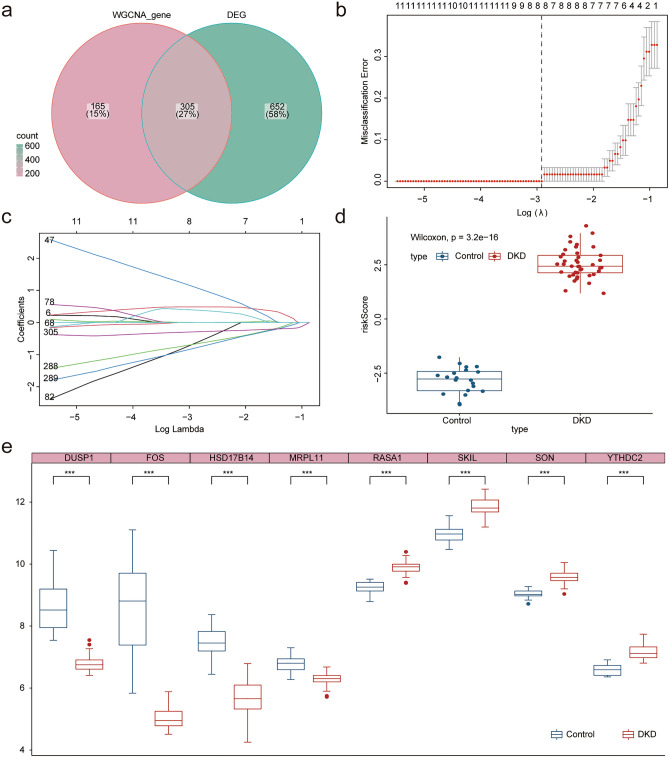
Construction of diagnostic model related to ferroptosis. (**a**) WGCNA obtained ferroptosis and diagnosis related gene module (WGCNA_gene) and intersected with DEGs. (**b**) The tuning parameters of LASSO model were selected by tenfold cross-validation. (**c**) LASSO coefficient spectrum of FRGs. The dotted line represents the selected value after 10 cross-validations. Finally, a ferroptosis-related diagnostic model containing eight genes was obtained. (**d**) Box chart shows the difference in risk scores between the DKD samples and the normal kidney tissue samples (Control). (**e**) Box diagram shows the differential expression of eight genes in the ferroptosis-related diagnostic model between the two groups (*p* < 0.05 was considered statistically significant).

**Figure 6:**
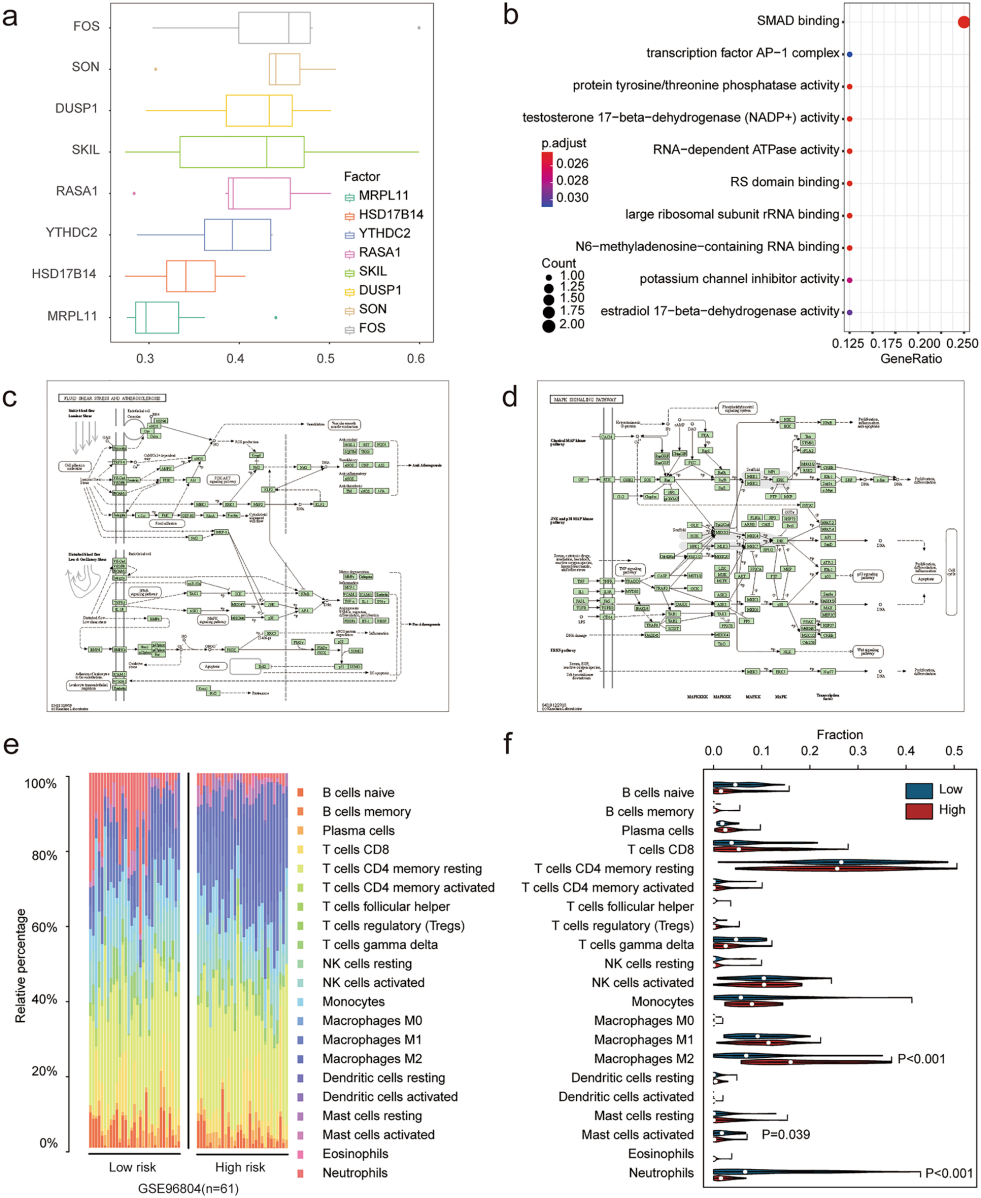
Gene function analysis of ferroptosis-related diagnostic model. (**a**) Friend analysis showed that FOS had the highest correlation with the other seven genes. (**b**) GO functional analysis demonstrated the possible biological functions involved. (**c**) MAPK signaling pathway display in KEGG pathway analysis. (**d**) Fluid shear stress and atherosclerosis demonstration in KEGG pathway analysis. (**e**) Bar chart shows the proportion of 22 cells assessed in each sample based on CIBERSORT algorithm. (**f**) Violin diagram shows the difference in distribution of 22 cells evaluated by CIBERSORT algorithm between high and low risk groups. Red represents the high-risk group and blue represents the low-risk group (*p* < 0.05 was considered statistically significant).

In order to construct a more accurate and reliable diagnosis model of DKD based on FRGs, we used WGCNA co-expression network analysis to search for gene modules related to ferroptosis and DKD. We obtained 10 consensus modules according to the results of the WGCNA. Next, we analyzed the correlation between the gene module and phenotypic data based on DKD and the enrichment score of ferroptosis. The results showed that the blue module (3,788) was positively correlated with DKD and negatively correlated with ferroptosis, while the green (576), green-yellow (114), and yellow modules (1237) were positively contrary (Fig. 4a–e). We obtained the key genes of each module with a correlation coefficient of 0.8 as the threshold and intersected with the differential genes. Finally, 305 ferroptosis gene sets related to DKD diagnosis were obtained (Fig. 5a). A diagnostic model was constructed by LASSO regression using the GLMNet package (Fig. 5b,c). Finally, we constructed a diagnostic model for DKD using eight FRGs (SKIL, RASA1, YTHDC2, SON, MRPL11, HSD17B14, DUSP1 and FOS), and calculated the risk score of each sample. The results showed that the risk score in the DKD group was significantly higher (Fig. 5d,e). We then analyzed the functions of these eight FRGs. Friend analysis showed that the FOS gene had the highest correlation with the other model genes (Fig. 6a), which may play more important role in disease. GO functional analysis indicated that multiple enzyme activities and transcriptional processes (Fig. 6b) may be involved (see Supplementary Table S4 online). KEGG^11,12^ functional analysis suggested that the MAPK signaling pathway, fluid shear stress, and atherosclerosis pathways were enriched (Fig. 6c,d) (see Supplementary Table S5 online). Immune infiltration is closely related to the development of DKD. In order to explore the relationship between ferroptosis and immune infiltration in DKD, we evaluated the immune cell infiltration of all samples through 22 kinds of immune cell gene sets using the CIBERSORT algorithm (Fig. 6e). The samples were divided into high and low risk groups according to the median risk score (low-risk group: 31; high-risk group: 30). We found that in the high-risk group, the expression of macrophages M2 increased significantly, whereas the expression of neutrophils and mast cells activated decreased significantly (Fig. 6f). Ferroptosis may affect the immune infiltration of Macrophages M2, Neutrophils and Mast cells activated immune cells in DKD.

**Figure 7:**
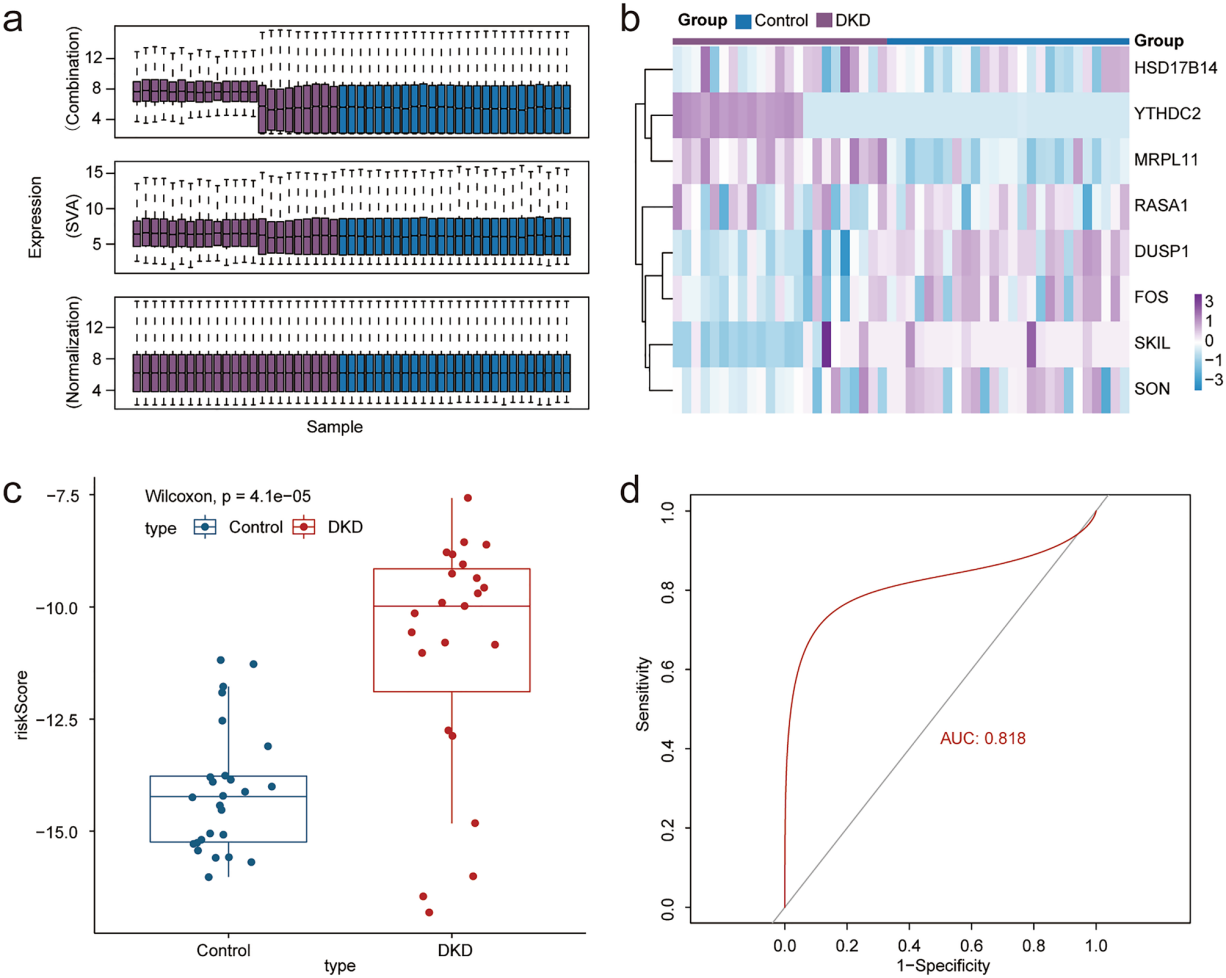
Model validation. (**a**) Data sets GSE30566, GSE99339 and GSE30528 were combined, batch removed and normalized. (**b**) Heat map shows the difference in the expression of model genes between the two groups. In the legend, purple represents DKD sample and dark blue represents normal kidney tissue sample (Control). (**c**) Box plot shows that the risk score in DKD group is significantly higher than that in control group after calculating the risk score of the new data set sample according to the model (*p* < 0.05 was considered statistically significant). (**d**) ROC curve confirmed that the diagnostic model still had high accuracy (AUC = 0.818), confirming that the prediction effect of the model is stable and repeatable.

The GSE30566, GSE99339, and GSE30528 datasets were used to verify the effectiveness of the prognostic model. After the de-batch effect and normalization processing, we obtained the verification data set (normal group 26; DKD group 23) (Fig. 7a). The risk score for each sample in the dataset was calculated based on the correlation coefficient of our prognostic model. The box chart showed that the risk score of the DKD group is significantly higher (Fig. 7b). We further used ROC curve and calibration to verify the accuracy of the model, and the results showed that the ferroptosis-related prognostic model still had a high diagnostic value. At the same time, the heat map indicated that there were significant differences in the expression of model genes in the test set (AUC = 0.818) (Fig. 7c–d). All results proved that the prognostic model we constructed was accurate and repeatable.

### Analysis of subtypes of ferroptosis

**Figure 8:**
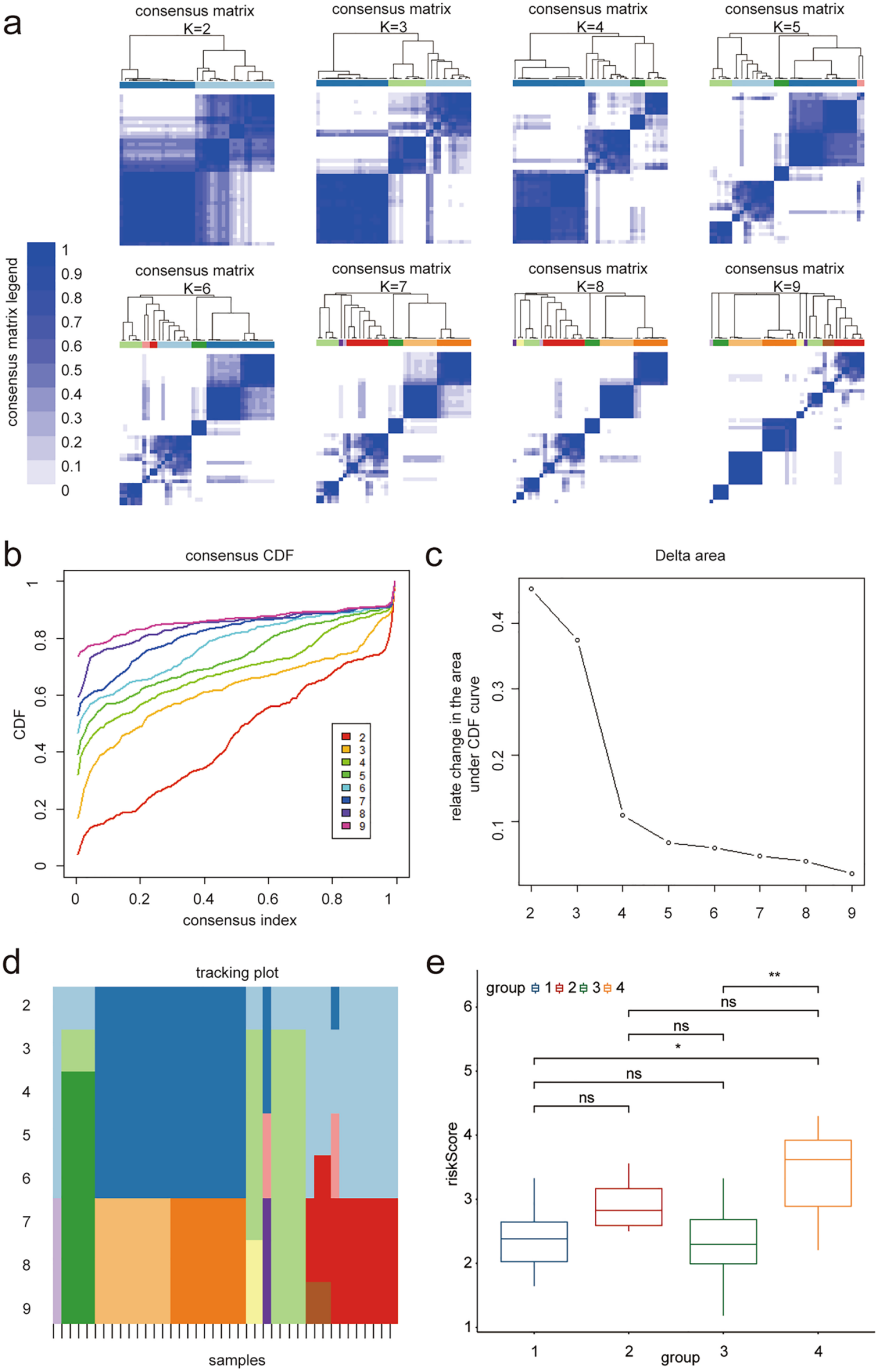
Ferroptosis subtypes. (**a**) The best immune classification was analyzed by consistency clustering analysis, matrix heat map is showed according to K from 2 to 9. Both rows and columns of the matrix represent samples. The consistency matrix clustering value is showed from 0 (impossible clustering together) to 1 (always clustering together) with white to dark blue. The consistency matrix is arranged according to the consistency classification (the tree map above the heat map). The bar between the tree map and the heat map is the category. (**b**) Consistent cumulative Distribution Function (CDF) graph: This graph shows the cumulative distribution function with different values of K, and CDF reaches an approximate maximum value when K = 4. (**c**) Incremental area of variables: when K = 4, the area under the CDF curve tends to be stable, and when K = 5, the relative change of CDF decreases significantly. (**d**) Tracking graph: The black stripe at the bottom of this graph represents the sample, which shows the classification of the sample when K is taken with different values. Different color blocks represent different classifications. The tracer map shows that the grouping is clear when K = 4. (**e**) Boxplot shows the differences of risk scores in different groups according to the four ferroptosis subgroups. The results showed that patients with type four had significantly higher risk scores while type 1 and type 3 had lower risk scores (*p* < 0.05 was considered statistically significant).

We used previously obtained FRGs to construct a molecular subtype model to further study the molecular subtypes of ferroptosis in DKD. According to the expression matrix of FRGs, all cases were analyzed using consistent cluster analysis to determine their potential ferroptosis types using the ConsensusClusterPlus package. The results showed four different ferroptosis types after 1000 repeated sampling (Fig. 8a–d), and the risk score of type four was significantly higher than that of the other groups (Fig. 8e). This may provide a theoretical basis for exploring personalized treatment measures for DKD according to the subtypes of ferroptosis in the future.

## Discussion

DM and its complications pose a major public health issue. The updated prevalence of diabetes in adults is 12.4% in China^13^. DKD, one of the main chronic complications of DM, is currently the main cause of renal replacement therapy^14^. As the concrete mechanism of DKD is unclear, there are no effective medicines or methods to prevent ESRD. Ferroptosis is an iron-dependent cell death mode induced by the excessive accumulation of lipid peroxidation products^15^. ROS plays a key role in ferroptosis^16^. The discovery of ferroptosis has provided a new understanding of the pathogenesis of various diseases.

In the present study, we performed differential expression and principal component analysis. The results showed that the expression profiles were significantly different between the DKD and normal groups. Functional enrichment analyses based on DEGs showed that ferroptosis-related pathways were significantly different between the two groups. The ferroptosis enrichment degree in the DKD group was significantly lower than that in the normal group. GO enrichment analysis showed that the collagen-containing extracellular matrix and extracellular matrix were enriched, while the mitochondrial inner membrane and mitochondrial matrix were inhibited. Abnormal accumulation of extracellular matrix produced by endothelial cells and podocytes leads to thickening of the glomerular basement membrane, an early pathological change in DKD^17^. The prognostic performance of the ferroptosis-related gene set was verified using the ROC curve analysis, which indicated that the model had a good prognostic value. Immune cell infiltration was further analyzed between the high- and low-risk groups. The results showed that macrophage M2 increased significantly in the high-risk group. This may be due to an overactivated immune response in patients with DKD. These results suggest that immunotherapy may be a therapeutic target for DKD.

Eight key FRGs (SKIL, RASA1, YTHDC2, SON, MRPL11, HSD17B14, DUSP1and FOS) were identified, and their validity in predicting the prognosis of DKD was analyzed. SKIL (also known as SnoN) is a regulator of the transforming growth factor-β (TGF-β) signaling pathway, which acts as an antifibrotic factor in the pathological process of DKD^18^. SKIL gene depletion prompted epithelial- mesenchymal transited into renal tubular cells in the condition of high glucose. Loss of SKIL expression appears to exacerbate progressive renal fibrosis in DKD^19^. Li et al.^20^ found that high glucose induced the downregulation of SKIL through the TGF-β1/Smad signaling pathway in human renal tubule epithelial cells. Bone morphogenetic protein-7 (BMP-7) ameliorates renal fibrosis by increasing the expression of SKIL in renal tubular epithelial cells^21^. However, the relationship between ferroptosis and SKIL in DKD remains unknown. RASA1 is a RasGAP signaling scaffold protein involved in various physiological processes, such as cell proliferation, differentiation, and apoptosis^22^. RASA1 inhibits renal tissue fibrosis by reducing myofibroblasts proliferation^23^. In kidney carcinoma, RASA1 reduces miR-223-3p expression to inhibit the proliferation and differentiation of renal cell carcinoma^24^. YT521-B homology domain containing 2(YTHDC2) is an m6A reader that expedites messenger ribonucleic acid (mRNA) decay. YTHDC2 increases the translation efficiency of target genes and reduces their mRNA abundance^25^. Ma et al.^26^ found that YTHDC2 is a powerful inducer of ferroptosis and that increasing YTHDC2 is an alternative therapy for lung adenocarcinoma targeting ferroptosis. SON is a ubiquitously expressed and evolutionarily conserved DNA and RNA binding protein localized in nuclear speckles. SON is involved in multiple cellular processes, including transcription, RNA splicing, and gene repression, which regulate the cell cycle and preserve stem cells^27,28^. Based on the above mechanism, SON plays a role in various diseases such as cancer, influenza, and hepatitis^29^. Overexpression of SON is involved in aberrant transcriptional initiation in leukemia^30^. MRPL11 contribute to protein synthesis as a mitochondrial ribosomal protein within the mitochondria. It mediates aerobic energy conversion through the oxidative phosphorylation system to affect the pathophysiological processes of various tumors^31,32^. The protein-coding variants of the hydroxysteroid 17-β dehydrogenase 14 gene (HSD17B14) can prevent the progression of type 1 DM to ESRD^33^. Dual-specificity phosphatase 1 (DUSP1), a regulator of the MAPK family, is associated with various pathological changes in the kidney, including renal hypertrophy, renal fibrosis, and glomerular apoptosis. Ge et al.^34^ proved that DUSP1 is involved in renal fibrosis in DKD through the miR-324-3p/DUSP1 axis. Another study demonstrated that DUSP1 could release DKD by targeting the JNK-Mff-mitochondrial fission pathways^35^. Researchers have found that DUSP1 can inhibit autophagy-dependent ferroptosis in human pancreatic cancer cells^36^. FOS was identified to play an important role in various kidney diseases such as membranous nephropathy^37^, immunoglobulin A nephropathy^38^, and chronic glomerulonephritis^39^. All FRGs have not been fully illustrated in the development of DKD. Further experiments are required to verify the functions of the key genes in DKD.

KEGG pathway enrichment analysis revealed that the MAPK signaling pathway was significantly enriched. The MAPK signaling pathway plays a crucial role in various physiological processes such as proliferation, differentiation, and metastasis^40,41^. It is involved in ferroptosis as a regulator of oxidative stress, which regulates signal transduction in a ROS-induced manner^42^. Poursaitidis^43^ showed that the inhibition of MAPK signaling protected lung cancer cells against ferroptosis. Wen-Tsan Chang^44^ demonstrated that the drug could induce hepatocellular carcinoma cell death through the MAPK pathway in the form of ferroptosis. In acute myeloid leukemia cells, inhibition of the MAPK pathway can render acute myeloid leukemia cells insensitive to ferroptosis^45^. Nevertheless, how FRGs affect the pathophysiological process of DKD through the MAPK pathway needs to be fully studied.

Our study has some limitations. First, only diabetic glomerular tissue samples were included, which may have led to one-sided results and selection bias. Therefore, in future studies, it will be necessary to improve the detection capability by integrating data from multiple tissue samples. Second, the sample size was relatively small, which may have resulted in a false-positive rate. This will facilitate an increase in sample size for further validation. Third, owing to the lack of pathological specimens of DKD in clinical settings, we were unable to assess the associations between risk indicators and pathological subtypes. In future studies, more pathological subtypes of DKD are needed to conduct further analyses. Fourth, our results are based on bioinformatic analysis, therefore, require further in vitro and in vivo verification. Fifth, our screening method can be used for gene screening in terms of its diagnostic value, phenotypic module clustering, differential expression and co-expression analysis, and clinical predictive models. It also has good value in screening ferroptosis-related molecules with diagnostic value in DKD. Based on these analyses, there were only eight genes left; thus, it is impossible to further screen key genes by protein–protein interaction (PPI). In the follow-up study, we will consider using a PPI interaction network to screen molecules further. Sixth, in our study, the effect of the ferroptosis pathway on the upstream and downstream regulation mechanisms of DKD was not considered. In a follow-up study, we will further study the multi-level regulatory mechanism of ferroptosis on DKD at epigenetic, transcriptional, and post-transcriptional levels through various molecular experiments and bioinformatics methods.

While an effective treatment for DKD has not yet emerged, we integrated comprehensive bioinformatic analyses to identify the biological functions and pathways associated with ferroptosis in the development of DKD. We found eight ferroptosis genes (SKIL, RASA1, YTHDC2, SON, MRPL11, HSD17B14, DUSP1 and FOS) which might be serve a vital role in the pathogenesis of DKD. Our results may provide a novel methodology for DKD early diagnosis and targeted therapies. Further experimental studies are needed to confirm the function of ferroptosis in DKD.

## Methods

### Data collection and preprocessing

We extracted the gene expression profiles of DKD (GSE96804,GSE30566,GSE99339 and GSE30528) from the GEO database using the GEOquery package^46^. The GSE96804 dataset^47^ from Homo sapiens, based on the GPL17586 platform, contains 61 samples, including 20 normal glomerular and 41 diabetic glomerular tissues. All the samples were included in this study. The GSE30566 dataset^48^ from Homo sapiens based on the GPL571 platform contains 26 samples, including 13 normal glomerular and 13 normal renal tubular control; 13 normal glomerular samples were included in this study. The GSE30528 dataset^48^ from Homo sapiens, based on the GPL571 platform, contains 26 samples, including 13 normal glomerular and 9 diabetic glomerular tissues. All the samples were included in this study. The GSE99339 dataset^49^ was obtained from Homo sapiens, and the data platforms were GPL19109 and GPL19184. It contained 184 samples, including 13 diabetic glomerular tissues, all of which were included in this study. The original data of GSE30566 and GSE30528 were normalized and standardized, and GSE30566, GSE30528 and GSE99339 were combined as validation data sets. The data were normalized and de-batch processed using the sva package^50^ and standardized using the limma package^51^. Subsequently, 382 FRGs were obtained from the FerrDb^52^. Our study is based on open-source data; therefore, there are no ethical issues or conflicts of interest.

### Construction and verification of the LASSO model

Currently, LASSO regression is a commonly used machine learning algorithm for the construction of diagnostic models. Regularization was used to solve the occurrence of overfitting in the process of curve fitting and to improve the accuracy of the model. The model was built using the GLMnet package^53^ with a parameter set.seed (2), family = "binomial". The risk score of each sample is calculated by the sum of the expression values of all model genes multiplied by the corresponding correlation coefficients.

### Difference expression analysis

We used the limma package to calculate the differential expression of genes between the normal and DKD groups in the GEO microarray data with log fold change |logFC| > 0.5 and adj. *p* < 0.01 as the threshold. The genes were upregulated in the DKD group if logFC > 0.5, and downregulated if logFC < − 0.5. The results of the differential expression analysis are shown in the heat map using the R package pheatmap and the volcano map using the ggplot2 package^54^.

### Functional enrichment analysis

GO analysis was used to conduct large-scale functional enrichment, including biological process (BP), molecular function (MF), and cellular component (CC) analyses. The KEGG database stores the genomes, biological processes, diseases, and medical information. GO biological process and KEGG pathway enrichment analyses of the DEGs were performed using the R clusterProfiler package^55^. The critical value of FDR(adj. *p*) < 0.05 was considered statistically significant^55^.

To study the differences in biological processes among different groups, we used GSEA for enrichment analysis according to the logFC arrangement based on the GSE96804 profile. GSEA is a computational method used to analyze whether a particular gene set has a statistical difference between the two biological states. In our study, we used GSEA to explore the differences in pathways and biological processes of the samples in the datasets. The "msigdb.v7.0.symbols" gene set were downloaded from the MSigDB^56^ database for GSEA analysis.

In addition, the enrichment scores of related pathways in the MSigDB database were calculated according to the gene expression matrix of each sample using the GSVA method using the R-packet GSVA^57^. The differences in samples were screened using the limma package^51^. Enrichment items with statistically significant differences are shown in the heat map. According to the gene expression matrix of each sample, the enrichment scores of the FRGs were calculated using the ssGSEA method and are displayed in boxplots.

### Gene co-expression analysis

WGCNA^58^ aims to identify co-expressed gene modules, analyze core genes in the network, and explore the relationships between modules and phenotypes. First, the soft threshold was calculated using the pickSoftTreshold function, and five was the best soft threshold. We then built a scale-free network based on the soft threshold. Next, a topology matrix and hierarchical clustering were constructed. We dynamically cut and identified the gene module and calculated the eigengenes, and the number of genes in each module was at least 50. The correlation between modules was constructed according to the eigengenes of the modules, and hierarchical clustering was performed. The modules were merged again with a correlation of more than 0.7, and finally, 10 modules were obtained. The correlations between modules and clinical features were explored using Pearson’s correlation analysis.

### Consistent cluster analysis

Consistent clustering is a method that can be used to determine the members and number of possible clusters in a dataset (microarray gene expression). In order to distinguish different subtypes of DKD, we carried out a consensus clustering analysis related to FRGs in the DKD group in the GSE96804 dataset using "ConsensusClusterPlus" R package^59^. In this process, the number of clusters was set between 2 and 10; 80% of the samples were taken each time and calculated 100 times, clusterAlg = "hc” and distance = "euclidean."

### Immune infiltration (CIBERSORT)

CIBERSORT^60^ is an algorithm for deconvolution of the transcriptome expression matrix, according to the principle of linear support vector regression, to estimate the composition and abundance of immune cells among mixed cells. The gene expression matrix data (TPM) were uploaded to CIBERSORT, combined with the LM22 gene matrix, and an immune cell infiltration matrix with filtration (*p* < 0.05) was obtained. A bar chart was drawn using the ggplot2 package in R language to show the distribution of 22 immune cell infiltrations in each sample.

### Statistical analyses

All data calculations and statistical analysis were conducted using R programming (https://www.r-project.org/, 4.0.2 version). For two groups of continuous variables, the statistical difference in normal distribution variables was estimated using an independent Student’s t-test, and non-normally distributed variables were analyzed using the Mann–Whitney U test (Wilcoxon rank sum test). A two-tailed *p* < 0.05 was considered statistically significant.

## Supplementary Information

Supplementary Figures.

Supplementary Tables.

## Data Availability

The original contributions presented in the study are included in the article/Supplementary Material. Further inquiries can be directed to the corresponding author.
