# Supplementary material for: Identification of ferroptosis-related genes and pathways in diabetic kidney disease using bioinformatics analysis
Source: Sci Rep. 2022 Dec 30;12:22613. doi: 10.1038/s41598-022-26495-2 (PMC9803720; doi:10.1038/s41598-022-26495-2)

Supplementary figure 1:

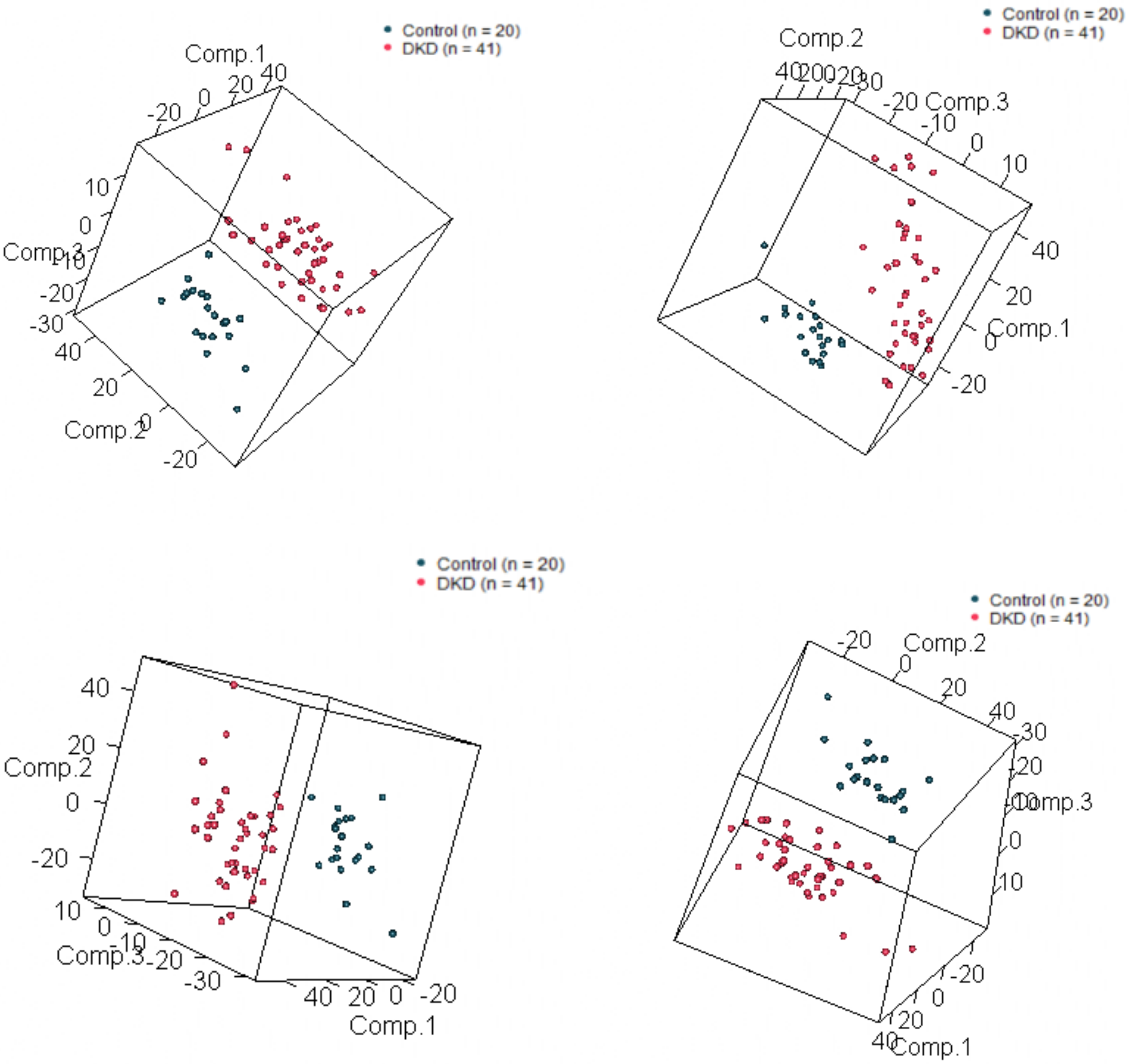

Supplementary figure 2:

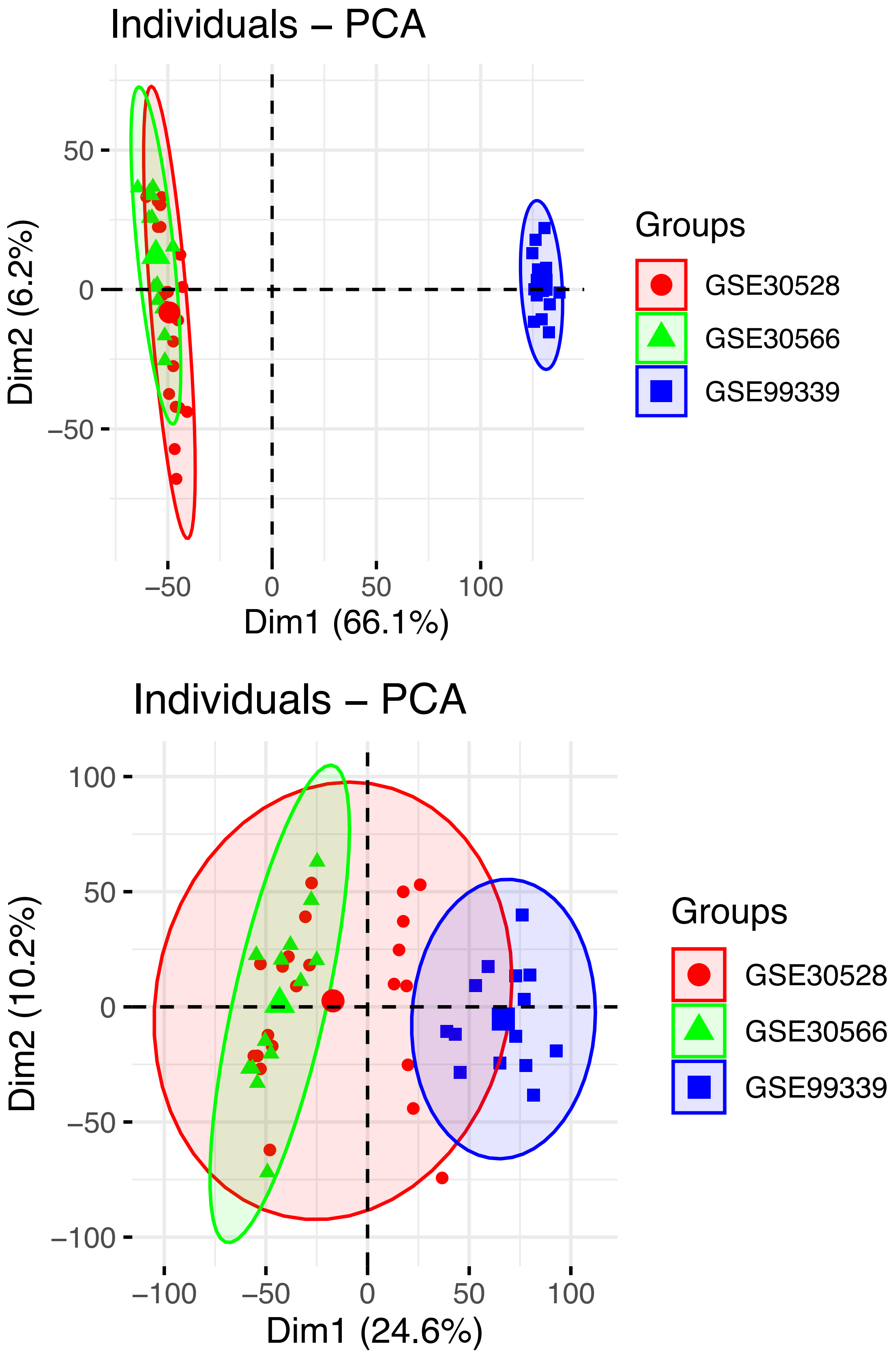

Supplementary figure 3:

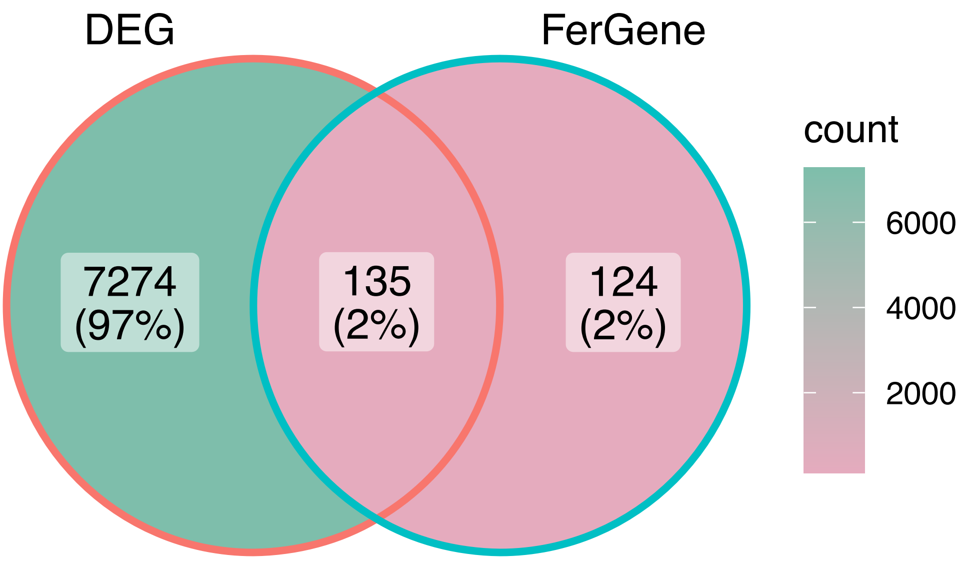

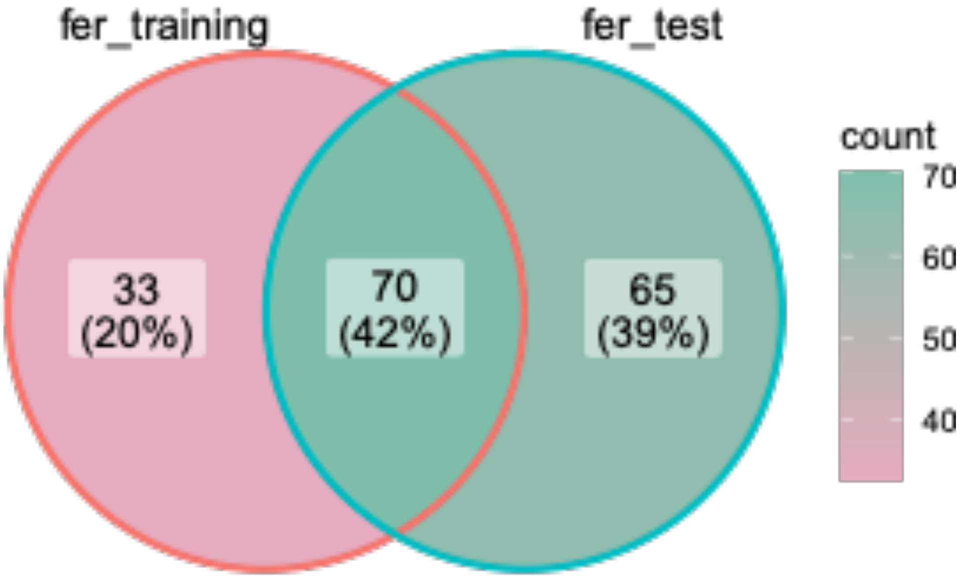

Supplementary figure 4:

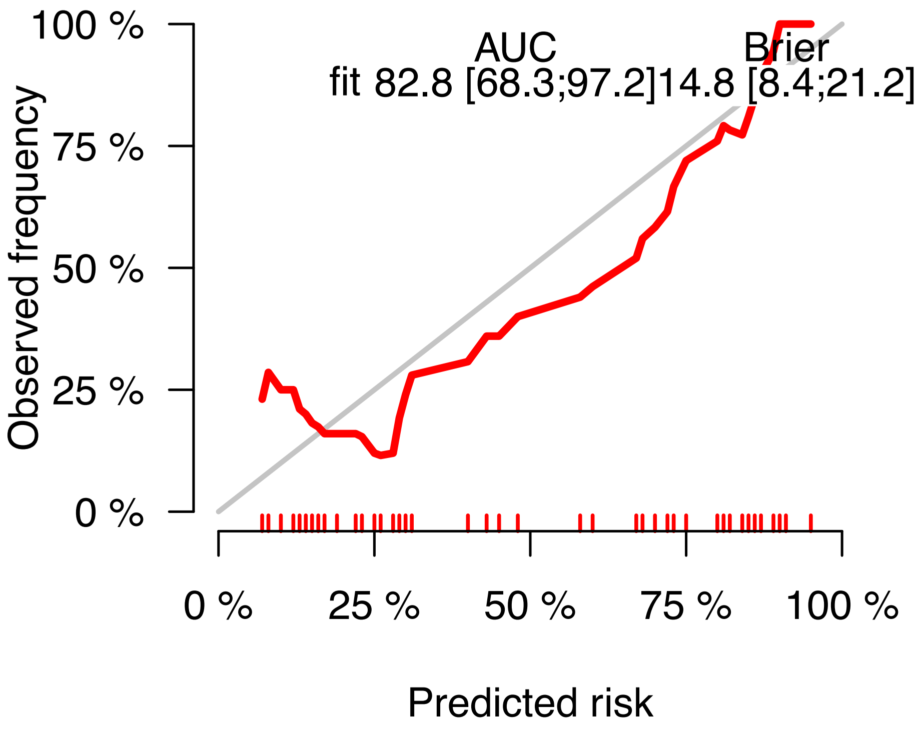

Supplementary figure 5:

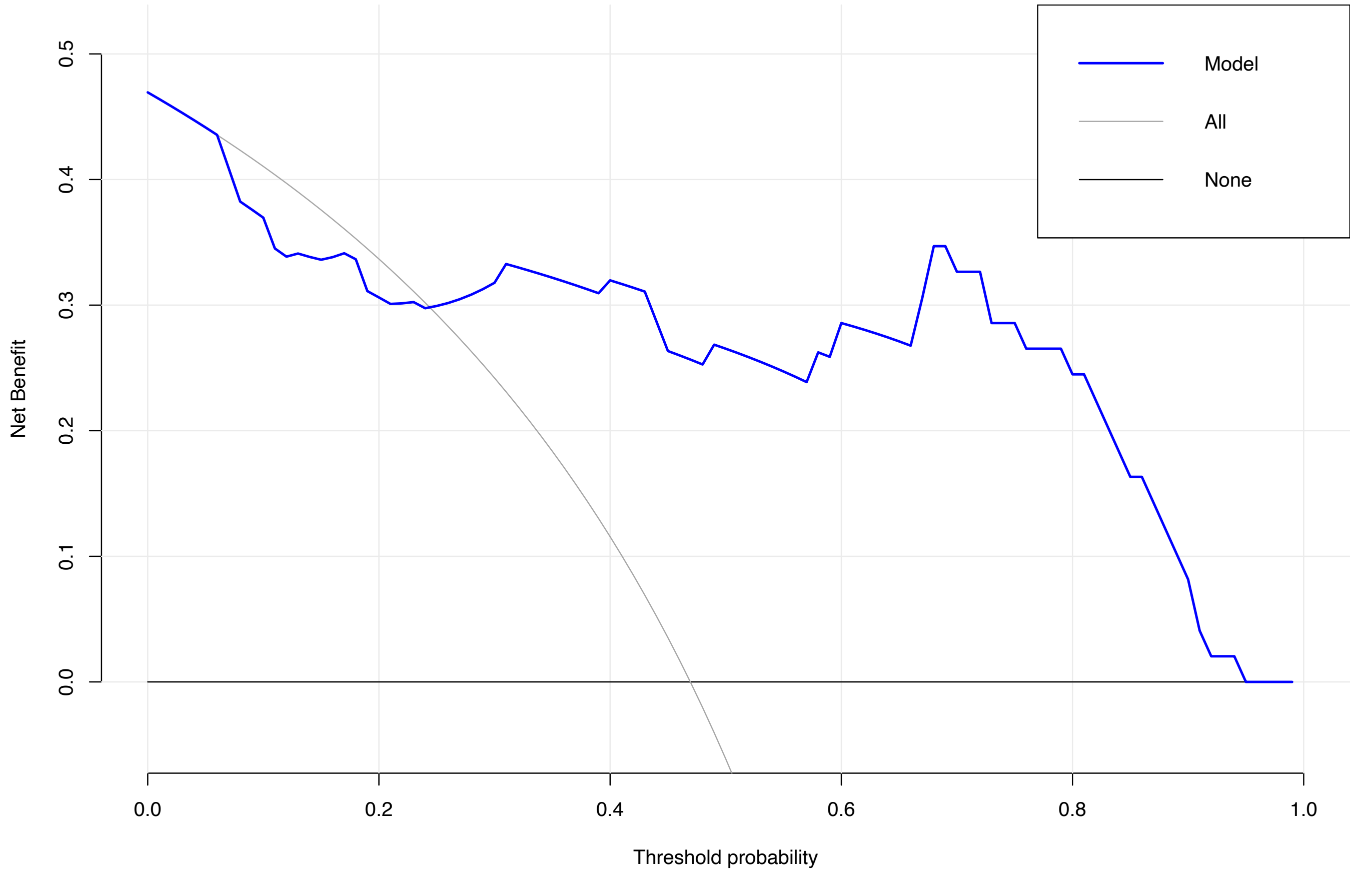

Supplementary figure 6:

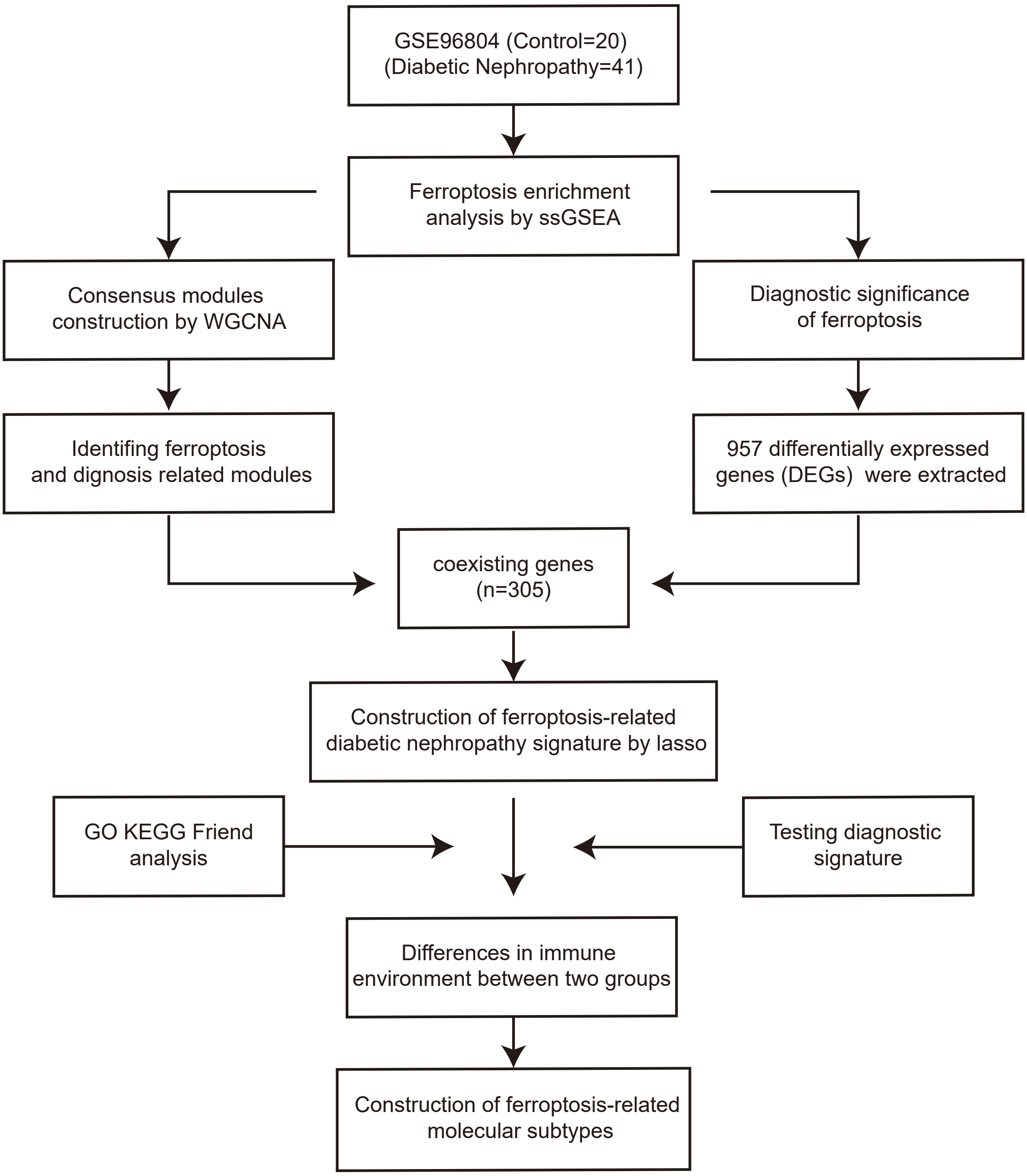

Supplement: Supplementary file 1 — Supplementary Figures. [file 41598_2022_26495_MOESM1_ESM.pdf]
