# Supplementary material for: Identification of ferroptosis-related genes and pathways in diabetic kidney disease using bioinformatics analysis
Source: Sci Rep. 2022 Dec 30;12:22613. doi: 10.1038/s41598-022-26495-2 (PMC9803720; doi:10.1038/s41598-022-26495-2)
Supplement: Supplementary file 2 — Supplementary Tables. [file 41598_2022_26495_MOESM2_ESM.pdf]

Supplementary tables

Table S1 Pathway enrichment analysis by GSVA analysis

Description	logFC	adjust-p
STANHILL_HRAS_TRANSFROMATION_UP	-1.15777	2.26E-23
GO_TRANSCRIPTION_FACTOR_AP_1_COMPLEX	-1.15065	1.18E-18
GSE7596_AKT_TRANSD_VS_CTRL_CD4_TCONV_WITH_TGFB_UP	0.492108	1.26E-14
GO_EPIBOLY	0.612853	2.88E-12
HUMMERICH_MALIGNANT_SKIN_TUMOR_DN	-0.63053	6.15E-12
GO_PML_BODY_ORGANIZATION	0.763429	6.15E-12
GALE_APL_WITH_FLT3_MUTATED_UP	0.484234	6.15E-12
GSE34156_UNTREATED_VS_6H_NOD2_LIGAND_TREATED_MONOCYTE_UP	-0.42947	6.15E-12
GSE39820_CTRL_VS_TGFBETA3_IL6_IL23A_CD4_TCELL_UP	0.441666	7.36E-12
GO_PORIN_ACTIVITY	-1.17032	1.06E-11

Table S2 GSEA enrichment analysis of GO function of DEGs in DKD

ID	Description	NES	adjust-p
GO:0062023	collagen-containing extracellular matrix	2.316283	0.02322
GO:0031012	extracellular matrix	2.233551	0.02322
GO:0005788	endoplasmic reticulum lumen	1.833626	0.02322
GO:0005925	focal adhesion	1.767585	0.02322
GO:0030055	cell-substrate junction	1.755424	0.02322
GO:0005681	spliceosomal complex	1.635317	0.02322
GO:0000781	chromosome, telomeric region	1.631534	0.02322
GO:0098687	chromosomal region	1.538351	0.02322
GO:0016607	nuclear speck	1.511965	0.02322
GO:0030426	growth cone	1.511335	0.029127
GO:0030427	site of polarized growth	1.507831	0.029127
GO:0034399	nuclear periphery	1.490349	0.044337
GO:0000793	condensed chromosome	1.48673	0.029127
GO:0030496	midbody	1.486664	0.044337
GO:0000775	chromosome, centromeric region	1.474894	0.044337
GO:0030027	lamellipodium	1.385593	0.04938
GO:0031252	cell leading edge	1.373402	0.030283
GO:0015629	actin cytoskeleton	1.363185	0.02322
GO:0005911	cell-cell junction	1.308235	0.046035
GO:0000228	nuclear chromosome	1.301941	0.031136
GO:0098797	plasma membrane protein complex	1.259755	0.046516
GO:0031253	cell projection membrane	-1.3663	0.04449
GO:0045177	apical part of cell	-1.46694	0.02322
GO:0019867	outer membrane	-1.52087	0.042711
GO:0016324	apical plasma membrane	-1.53072	0.02322
GO:0031968	organelle outer membrane	-1.54122	0.042711
GO:0005741	mitochondrial outer membrane	-1.65908	0.02322

GO:0098862	cluster of actin-based cell projections	-1.71711	0.02322
GO:0019866	organelle inner membrane	-1.86844	0.02322
GO:0031966	mitochondrial membrane	-1.89095	0.02322
GO:0005740	mitochondrial envelope	-1.90933	0.02322
GO:0098798	mitochondrial protein complex	-1.94669	0.02322
GO:0005743	mitochondrial inner membrane	-1.96784	0.02322
GO:0005759	mitochondrial matrix	-2.30586	0.02322

Table S3 GSEA enrichment analysis of KEGG pathway of DEGs in diabetic nephropathy

**ID**	**Description**	**NES**	**adjust-p**
hsa04510	Focal adhesion	2.013966	0.010433
hsa05206	MicroRNAs in cancer	1.854934	0.010433
hsa05205	Proteoglycans in cancer	1.728944	0.010433
hsa04151	PI3K-Akt signaling pathway	1.716662	0.010433
hsa05165	Human papillomavirus infection	1.706475	0.010433
hsa05168	Herpes simplex virus 1 infection	1.597501	0.010433
hsa05164	Influenza A	1.566219	0.010433
hsa04060	Cytokine-cytokine receptor interaction	1.549929	0.010433
hsa04621	NOD-like receptor signaling pathway	1.534997	0.010433
hsa04810	Regulation of actin cytoskeleton	1.51966	0.016389
hsa05169	Epstein-Barr virus infection	1.516306	0.010433
hsa05171	Coronavirus disease - COVID-19	1.481894	0.010433
hsa05131	Shigellosis	1.471281	0.021177
hsa05160	Hepatitis C	1.451674	0.015811
hsa05203	Viral carcinogenesis	1.445467	0.010433
hsa04218	Cellular senescence	1.424482	0.015811
hsa04062	Chemokine signaling pathway	1.420955	0.010433
hsa04310	Wnt signaling pathway	1.392238	0.030614
hsa05202	Transcriptional misregulation in cancer	1.36693	0.020726
hsa05200	Pathways in cancer	1.268244	0.033315
hsa04714	Thermogenesis	-1.48649	0.020726
hsa04932	Non-alcoholic fatty liver disease	-1.60892	0.010433
hsa05208	Chemical carcinogenesis - reactive oxygen species	-1.79856	0.010433

Table S4 GO functional analysis of diagnostic markers

ID	Description	adjust-p
GO:0035976	transcription factor AP-1 complex	0.03082
GO:0046332	SMAD binding	0.024383
GO:0008330	protein tyrosine/threonine phosphatase activity	0.024383
GO:0047045	testosterone 17-beta-dehydrogenase (NADP+) activity	0.024383
GO:0008186	RNA-dependent ATPase activity	0.024383
GO:0050733	RS domain binding	0.024383
GO:0070180	large ribosomal subunit rRNA binding	0.024383
GO:1990247	N6-methyladenosine-containing RNA binding	0.024383
GO:0019870	potassium channel inhibitor activity	0.02742
GO:0004303	estradiol 17-beta-dehydrogenase activity	0.029778
GO:0017017	MAP kinase tyrosine/serine/threonine phosphatase activity	0.031661
GO:0033549	MAP kinase phosphatase activity	0.033197
GO:0034458	3'-5' RNA helicase activity	0.038515
GO:0070412	R-SMAD binding	0.043003
GO:0051019	mitogen-activated protein kinase binding	0.045114
GO:0033764	steroid dehydrogenase activity, acting on the CH-OH group of donors, NAD or NADP as acceptor	0.046937
GO:0000979	RNA polymerase II core promoter sequence-specific DNA binding	0.04845
GO:0016229	steroid dehydrogenase activity	0.04845
GO:0008200	ion channel inhibitor activity	0.04845
GO:0016248	channel inhibitor activity	0.04845
GO:0001784	phosphotyrosine residue binding	0.04845
GO:0001046	core promoter sequence-specific DNA binding	0.049502
GO:0008138	protein tyrosine/serine/threonine phosphatase activity	0.049502

Table S5 KEGG analysis of diagnostic markers

ID	Description	adjust-p
hsa04010	MAPK signaling pathway	0.022311
hsa05418	Fluid shear stress and atherosclerosis	0.070435
